# Edge-Centric Embeddings of Digraphs: Properties and Stability Under Sparsification

**DOI:** 10.3390/e27030304

**Published:** 2025-03-14

**Authors:** Ahmed Begga, Francisco Escolano Ruiz, Miguel Ángel Lozano

**Affiliations:** Department of Computer Science and Artificial Intelligence, University of Alicante, 03690 Alicante, Spain; sco@ua.es (F.E.R.); malozano@ua.es (M.Á.L.)

**Keywords:** edge embedding, line digraph, edge mining, digraph sparsification, graph neural networks, maximum entropy

## Abstract

In this paper, we define and characterize the embedding of edges and higher-order entities in directed graphs (digraphs) and relate these embeddings to those of nodes. Our edge-centric approach consists of the following: (a) Embedding line digraphs (or their iterated versions); (b) Exploiting the rank properties of these embeddings to show that edge/path similarity can be posed as a linear combination of node similarities; (c) Solving scalability issues through digraph sparsification; (d) Evaluating the performance of these embeddings for classification and clustering. We commence by identifying the motive behind the need for edge-centric approaches. Then we proceed to introduce all the elements of the approach, and finally, we validate it. Our edge-centric embedding entails a top-down mining of links, instead of inferring them from the similarities of node embeddings. This analysis is key to discovering inter-subgraph links that hold the whole graph connected, i.e., central edges. Using directed graphs (digraphs) allows us to cluster edge-like hubs and authorities. In addition, since directed edges inherit their labels from destination (origin) nodes, their embedding provides a proxy representation for node classification and clustering as well. This representation is obtained by embedding the line digraph of the original one. The line digraph provides nice formal properties with respect to the original graph; in particular, it produces more entropic latent spaces. With these properties at hand, we can relate edge embeddings to node embeddings. The main contribution of this paper is to set and prove the *linearity theorem*, which poses each element of the transition matrix for an edge embedding as a linear combination of the elements of the transition matrix for the node embedding. As a result, the *rank preservation property* explains why embedding the line digraph and using the labels of the destination nodes provides better classification and clustering performances than embedding the nodes of the original graph. In other words, we do not only facilitate edge mining but enforce node classification and clustering. However, computing the line digraph is challenging, and a sparsification strategy is implemented for the sake of scalability. Our experimental results show that the line digraph representation of the sparsified input graph is quite stable as we increase the sparsification level, and also that it outperforms the original (node-centric) representation. For the sake of simplicity, our theorem relies on node2vec-like (factorization) embeddings. However, we also include several experiments showing how line digraphs may improve the performance of Graph Neural Networks (GNNs), also following the principle of maximum entropy.

## 1. Introduction

Graph Neural Networks (GNNs) [1,2,3,4] are of pivotal importance for the advancement of AI in structural domains such as molecular chemistry, social-network analysis [5], and even algorithmic reasoning [6,7]. GNNs are *node-centric*, since they exploit adjacency information to learn latent spaces where the nodal features are encoded for node classification and graph classification. Link/edge prediction, however, does usually rely on the latent representations of the nodes supposed to form an edge.

So far, capturing the latent spaces of edges has been an elusive problem. For instance, HOPE [8] exploits the powers of the adjacency matrix to assign asymmetric roles (source and target) to the nodes via a double embedding. This leads to edge prediction, but the resulting long-range similarity tends to diffuse local contexts, which results in poor node classification. The NERD method [9] overcomes the latter limitation by using alternating random walks, a standard method for estimating node centrality in directed graphs [10]. These walks provide, again, two embeddings: one for the source role of the node and another for its target role. The product of these embeddings provides a good performance in link prediction and graph reconstruction, and the concatenation of the embeddings is also competitive with node-centered methods in node-classification.

**Figure 1:**
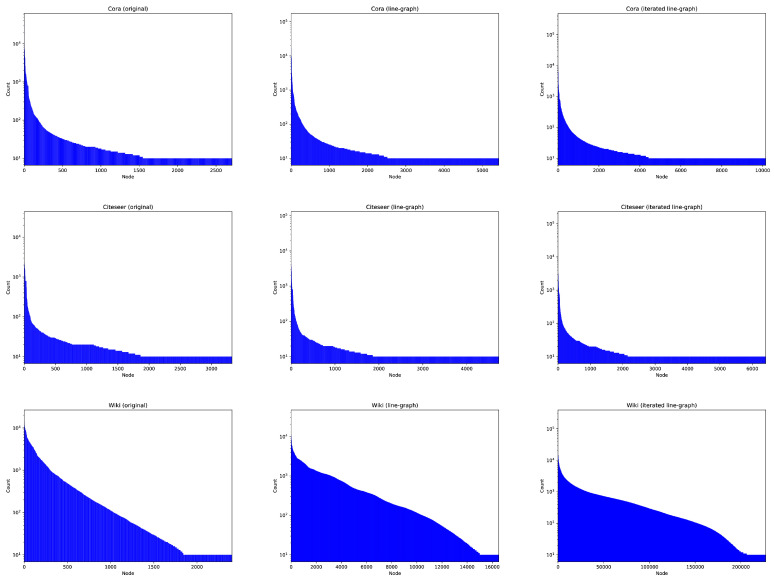
The Long-Tail Effect. Histogram of occurrence of each node in the random walks for Cora (**top**), Citeseer (**middle**) and Wiki (**bottom**) datasets, by using the original graph (**left**), the line graph (**center**), and the iterated line graph (**right**). *y* axis is represented in log scale.

However, the issues derived from addressing edge-related tasks such as link prediction from a node-centric angle have not been recognized until recently [11]. The degree distribution of most social networks follows a power law: a small number of nodes has a large number of neighbors, whereas most of the nodes have a small degree, and they are in the tail of the degree distribution. This is the so-called *long-tail effect*: link prediction of GNNs is greatly hindered by the tail node pairs since they share few neighbors. See, for instance, Figure 1-Left, where we plot the probabilities of reaching each node from a bunch of random walks for three social networks. Therein, the tail effect is quite visible: half of the nodes are unreached. However, in the Center and Right columns of Figure 1 we present more entropic distributions, thereby mitigating the long-tail effect to some extent, but how do we construct these distributions?

**Figure 2:**
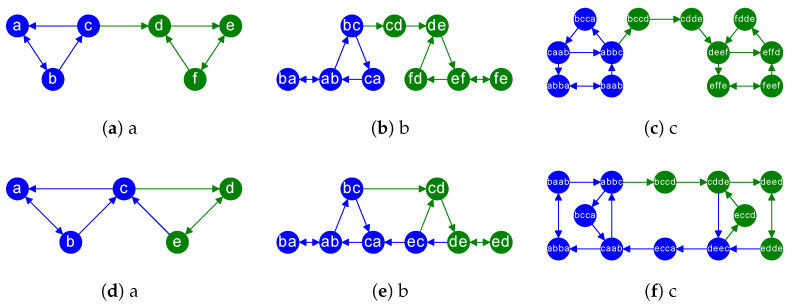
Examples of (**a**,**d**) original digraphs, (**b**,**e**) their line digraphs, and (**c**,**f**) their iterated line digraphs. The color of the nodes represents two different labels. Herein, edges are labeled using the label of the destination node. In other application domains may be more convenient to use the label of the source.

**Maximum Entropy Latent Spaces**. Instead of modifying the structure to increase the number of neighbors among under-represented nodes, as done in [11], we propose a more principled approach in this paper. In particular, we *turn our attention to the latent spaces of edges themselves*. Such spaces must produce similar codes for the edges having a similar structural role in the network. This requirement is even stronger when the networks encode causal relationships via directed edges. We show a couple of examples in Figure 2-Left column **(a)**,**(d)**, where there are two distinct roles: intra-community edges and inter-community edges. The nodes of intra-community edges have the same color (blue and green, respectively), whereas inter-communities have different colors in their nodes.

Then, in Figure 2-Center column **(b)**,**(e)**, we show a graph, say $\ell G=(V_{\ell },E_{\ell })$, where the nodes are the edges of the original graph G=(V,E), i.e., $V_{\ell }=E$. There is an edge $\left(ij,kl\right)\in E_{\ell }$ iff (i,j)∈E,(k,l)∈E and j=k. In other words, edges in ℓG, the so-called *line graph* of *G*, encode transitive relationships in *G*. As a result, paths and loops are shortened in the line graph. Since the edges of *G* are now the nodes of ℓG, we can apply a node-centric approach to ℓG in order to uncover the properties of the latent spaces of the edges in *G*. Independently of whether we use a node2vec-like embedding [12,13,14,15,16] or the embedding resulting from a state-of-the-art (SOTA) or more recent GNN [17,18,19,20,21], the subsequent latent space relies on how the structure constrains the random walks exploring it.

For instance, the Center and Right columns of Figure 2, show the line graphs of the graphs in the previous columns. A brief observation reveals that the line graph produces denser communities while preserving inter-class edges. Thus, shortening the paths and loops is respectful of the community structure of the graph: communities become redundant, but the inter-community information flow is preserved. In other words, random walks launched inside a given community visit it faster, but their probability of visiting another community is preserved with respect to the one in the original graph.

**Main Contribution**. Considering the above observations, and pointing out that ℓG has O(|E|) nodes instead of O(|V|), *in this paper we give spectral arguments showing that line graphs lead naturally to quasi-maximum entropy embeddings*.

Thus, this paper goes beyond the previous (instrumental) uses of line graphs. For instance, in the LGNN (line graph NN) model [22], line graph features complement node-based features for community detection. Similarly, in PRUNE [23] line graphs are implicitly computed via second-order proximity (considering the respective direct predecessors and successors of node pairs). Second-order proximity was introduced in LINE [14]. However, in [24] second-order proximity is used to bridge the microscopic scale of nodes with the mesoscopic community structure. This is interpreted in PRUNE via tri-factorization. Finally, GNNs have recently incorporated convolutions on both nodes and edges [25], and transfer learning [26] has been applied to node-centric approaches. In this latest regard, node-centric embeddings fail to capture transferable edges since they infer edges in a node-centric way and they are subject to the long-tail effect. We conjecture that modeling edges themselves would help to better transfer structural information.

**Organization of the paper**. The remainder of the paper is organized as follows. In Section 2, we introduce the main properties of line digraphs and uncover the formal relationship between nodal embeddings and the embedding of edges/paths. This relationship is linked to the rank property of the line digraph (the rank of a line digraph is the number of nodes of the original digraph), and this makes the difference in classification and clustering. Actually, large rank is an algebraic interpretation of maximum entropy [27]. In Section 3, we present our experimental results (edge classification and clustering), both in node2vec-like methods and GNNs, where we compute a proxy of the nodes classification/clustering. We show that this proxy not only outperforms the original (node-centric) classification and clustering but also has stable performance at different levels of sparsification. Finally, in Section 4, we sketch our conclusions and future work.

## 2. Method

### 2.1. Embedding Edges and Paths

This section presents the main theoretical contribution of this paper: the *linearity theorem* and its corollary, namely the *rank-preservation property*. We commence by defining line digraphs and introducing some of their formal properties. We focus our attention on two complementary concepts: (a) The preservation of the non-zero spectrum of the original digraph; (b) The relative density and diameter.

Given an input graph *G*, its line digraph ℓG tends to shorten the cycles in *G*, and this results in holding a small degree and diameter with respect to *G*. When we translate these facts to the respective transition matrices, we find out that running random walks in ℓG (instead of in *G*) results in the following: (a) Sampling a larger number of nodes; (b) Doing the sampling in a more efficient way (lower probability of returning to the origin); (c) Getting less oversampled nodes, which makes the representation less biased towards notable nodes, i.e., more entropic, thus reducing the long-tail effect.

With the above properties at hand, we prove (*linearity theorem*) that the similarity between two edges (entry of the *r*—the power of the transition matrix of ℓG) is a linear combination of the similarities between several pairs of nodes (entries of the transition matrix of *G*). Since the respective embeddings of the nodes of *G* and those of ℓG (the edges of *G*) rely on the weighted sums of these powers, we have that the matrix-to-factorize for ℓG tends to preserve its rank much better than that for *G* (*rank-preservation corollary*). This corollary explains why embedding the line digraph and using the labels of the destination nodes provides better classification and clustering performances than embedding the nodes of the original graph (we test this hypothesis in the experimental section).

Therefore, we contribute with theoretical results having important practical implications beyond explaining the link between the embedding of edges and that of nodes. In general, due to the *iterative property* of line digraphs, our rationale is extensible to line digraphs of line digraphs (path embedding), provided that there are enough computational resources to compute and store the *iterated digraph*. In this regard, we rely on digraph sparsification techniques to make this method scalable.

### 2.2. Line and Iterated Line Digraphs

**Similarity of** *G* **and** ℓG. Let G=(V,E) be a digraph with *n* nodes *V* and *m* edges *E*. Its line digraph $\ell G=(V_{\ell },E_{\ell })$ is a graph where the nodes $V_{\ell }=E$ are the edges of *G* and there is an edge between two nodes of $ij,kl\in V_{\ell }$ if j=k, i.e., if the edges (i,j),(k,l)∈E share the node j=k. Then, *G* and ℓG are encoded by their respective adjacency matrices which are related as follows:

(1) $$A_{G}=HT^{T},\;\;A_{\ell G}=T^{T}H\;,$$

where H is the n×m *incidence matrix of heads* with $H_{ij}=1$ if the node *i* is the head of the edge *j* and $H_{ij}=0$ otherwise, and T is the n×m *incidence matrix of tails*, with $T_{ij}=1$ if the node *i* is the tail of the edge *j* and $T_{ij}=0$ otherwise.

**Example**. Consider the Top-Left digraph (directed graph) in Figure 2. We have V={a,b,c,d,e,f} and E={(a,b),(b,a),(b,c),(c,a),(c,d),(d,e),(e,f),(f,d),(f,e)}. The edges are ordered in lexicographic order: $e_{1}=\left(a,b\right),e_{2}=\left(b,a\right),\ldots ,e_{9}=\left(f,e\right)$. Then, we have the following matrix product $A_{\ell G}=T^{T}H$:

(2) $$\left[\begin{array}{cccccc} 0 & 1 & 0 & 0 & 0 & 0\\ 1 & 0 & 0 & 0 & 0 & 0\\ 0 & 0 & 1 & 0 & 0 & 0\\ 1 & 0 & 0 & 0 & 0 & 0\\ 0 & 0 & 0 & 1 & 0 & 0\\ 0 & 0 & 0 & 0 & 1 & 0\\ 0 & 0 & 0 & 0 & 0 & 1\\ 0 & 0 & 0 & 1 & 0 & 0\\ 0 & 0 & 0 & 0 & 1 & 0 \end{array}\right]\cdot \left[\begin{array}{ccccccccc} 1 & 0 & 0 & 0 & 0 & 0 & 0 & 0 & 0\\ 0 & 1 & 1 & 0 & 0 & 0 & 0 & 0 & 0\\ 0 & 0 & 0 & 1 & 1 & 0 & 0 & 0 & 0\\ 0 & 0 & 0 & 0 & 0 & 1 & 0 & 0 & 0\\ 0 & 0 & 0 & 0 & 0 & 0 & 1 & 0 & 0\\ 0 & 0 & 0 & 0 & 0 & 0 & 0 & 1 & 1 \end{array}\right]=\left[\begin{array}{ccccccccc} 0 & 1 & 1 & 0 & 0 & 0 & 0 & 0 & 0\\ 1 & 0 & 0 & 0 & 0 & 0 & 0 & 0 & 0\\ 0 & 0 & 1 & 1 & 0 & 0 & 0 & 0 & 0\\ 1 & 0 & 0 & 0 & 0 & 0 & 0 & 0 & 0\\ 0 & 0 & 0 & 0 & 0 & 1 & 0 & 0 & 0\\ 0 & 0 & 0 & 0 & 0 & 0 & 1 & 0 & 0\\ 0 & 0 & 0 & 0 & 0 & 0 & 0 & 1 & 1\\ 0 & 0 & 0 & 0 & 0 & 1 & 0 & 0 & 0\\ 0 & 0 & 0 & 0 & 0 & 0 & 1 & 0 & 0 \end{array}\right]\;.$$

Note that the rows of $H^{T}$ correspond to the edges in *G* and the respective columns contain the target node of each edge. The first edges in ℓG are given by $H_{1:}^{T}\cdot T_{:2}=1$ and $H_{1:}^{T}\cdot T_{:3}=1$: (ab,ba) and (ab,bc) respectively. Then, we have $H_{2:}^{T}\cdot T_{:1}=1$ leading to (ba,ab) and $H_{3:}^{T}\cdot T_{:4}=1$ and $H_{3:}^{T}\cdot T_{:5}=1$ leading respectively to (bc,ca) and (bc,cd). Then, we obtain 12 edges in ℓG: the nodes of the digraph in Figure 2b.

An *iterated line digraph* $\ell^{p}G$, with p>1 is $\overset{p\;\mathrm{times}}{\overset{︷}{\ell \ell \ldots \ell }}G$. For instance, in Figure 2 we have that the digraph **(c)** is $\ell^{2}G$, that is, the line graph of ℓG: see digraph **(c)** with 12 nodes. Definitively, computing iterated line graphs allows us to understand the *latent spaces of directed paths* encoding causal chains.

**Coding Intuition: The Rank Property**. As shown in the example above, we have highlighted (in bold) the rows that are *unique*, i.e., those defining the algebraic rank of $A_{\ell G}$. We will build our *linearity theorem* on a result from [28]: rank$\left(A_{\ell G})=|V\right|$. However, at this point in the paper, the intuition that there are as many *different connectivity patterns in ℓG as nodes in G* is quite useful for the reader. Actually, this fact reveals the coding properties of ℓG wrt *G*. We highlight two basic consequences:

(a) We have that $\mathrm{rank}\left(A_{G}\right)\leq \mathrm{rank}\left(A_{\ell G}\right)=\left|V\right|$. Note that node2vec-like latent spaces are rooted in a SVD factorization (PCA) involving the adjacency matrix. As a result, the latent spaces of ℓG are, at least, *as entropic as* those of *G*: exactly |V| codewords are needed to explain the ways of linking the |E| nodes of ℓG.
(b) When it comes to GNNs, we observe a similar result. The foundational mechanism of a GNN is message passing: (i) Nodes are endowed with feature vectors; (ii) These vectors are propagated using the adjacency; (iii) The vectors received by each node are combined and projected on a learnable matrix. Having $\mathrm{rank}(A_{\ell G})=|V|$ implies that |V| of the |E| nodes in ℓG integrate their neighboring information in a different and orthogonal way. As a result, the subsequent latent spaces the nodes in ℓG are at least as entropic as those of the nodes in *G*. These spaces are actually less prone to the *over-smoothing problem* (hindering of the performance by decreasing the entropy of the coding due to repeated aggregation).

### 2.3. The Spectral Argument

The rank property is transferred to random walks as follows.

**Preservation of the spectrum of** *G* **in** ℓG. The line digraph ℓG is somewhat redundant with respect to *G*: the respective adjacencies $A_{G}$ and $A_{\ell G}$ are called *Flanders pair* in [29]; they have the same non-zero eigenvalues, with the same multiplicities, and also have the same number of distinct eigenvectors and generalized eigenvectors in each case associated with these non-zero eigenvalues (see also [30]). The transition matrices $P_{G}=D_{G}^{-1}A_{G}$ and $P_{\ell G}=D_{\ell G}^{-1}A_{\ell G}$ also share the same non-zero spectrum. This is key for understanding how random walks sample both *G* and ℓG. In particular, we have [31]: $tr\left(P_{\ell G}^{r}\right)=tr\left(P_{G}^{r}\right)\;\mathrm{for}\;\mathrm{all}\;r$ (equal traces for all powers of P).

**Transport Efficiency**. The condition $tr\left(P_{\ell G}^{r}\right)=tr\left(P_{G}^{r}\right)$ leads to the following expected probabilities of returning to the starting node: $E_{G}\left(r\right)=\frac{1}{n}tr\left(P_{G}^{r}\right)$ and $E_{\ell G}\left(r\right)=\frac{1}{m}tr\left(P_{\ell G}^{r}\right)$, where n=|V| and m=|E|. Borrowing the terminology from continuous-time random walks, $E_{G}\left(r\right)$ and $E_{\ell G}\left(r\right)$ quantify the *efficiency of the respective random walks* in terms of diffusing far from the origin (the smaller, the more efficient) [32]. Thus, ℓG is more efficient than *G*, and to some extent this mitigates the long-tail effect, as we show in Figure 1.

**Quasi-Maximum Entropy**. Maximal Entropy Random Walks [33] require that paths of a given length *r* are equiprobable. The condition $tr\left(P_{\ell G}^{r}\right)=tr\left(P_{G}^{r}\right)$ for all *r*, leads to $nE_{G}\left(r\right)=mE_{\ell G}\left(r\right)$, i.e., $E_{\ell G}\left(r\right)=\frac{n}{m}E_{\ell G}\left(r\right)$. Then, the denser *G* we have m≫n and $E_{\ell G}\left(r\right)\to 0$ (maximal efficiency). Under these conditions, we have Maximal Entropy for any cycle of any length. This is consistent with envision the ℓG as a quasi-entropy maximizers with respect to *G*: it maximizes the entropy of *G* subject to preserving the spectrum. Since the transition matrices $P_{G}$ and $P_{\ell G}$ share the same non-zero spectrum, they have an identical asymptotic behavior (same maximal eigenvalue).

**Density and diameter of** *G* **vs.** ℓG. Line digraphs hold small degree and diameter in relation to their large number of nodes *m* [28]. This means that when we are forced to place *m* nodes in ℓG with respect to the *n* nodes of *G*, ℓG (loosely) works as a complete digraph without forcing each node to be adjacent to all the others [34]. More formally, some line graphs are *almost Moore graphs*. For instance, if the order (sum of all nodes that can be reached from any node) of *G* is upper bounded [35] by $M_{\lambda_{1},D}\left(G\right)=\left(\lambda_{1}^{D+1}-1\right)/\left(\lambda_{1}-1\right)$, then the order of ℓG is upper bounded by $m\leq M_{\lambda_{1},D}\left(\ell G\right)=\left(\lambda_{1}^{D+2}-1\right)/\left(\lambda_{1}-1\right)$, where *D* is the diameter of *G*, and $\lambda_{1}$ is the leading eigenvalue of $A_{G}$ (and $A_{\ell G}$ since both matrices share the non-zero spectrum). Therefore, we have $O(\lambda_{1}^{D})$ vs. $O(\lambda_{1}^{D+1})$ bounds. These bounds control, respectively, the reachability of the nodes in both graphs. Again, the largest reachability bound of ℓG allows us to mitigate the long-tail effect.

### 2.4. The Rank Property of LDEs

Since node-centric graph embeddings can be seen as factorizing a matrix S relying on the powers of P, the transition matrix [36], the above properties suggest a deeper analysis of $S_{G}$ vs. $S_{\ell G}$. (The respective *matrices-to-factorize* for *G* and ℓG). In particular, the preservation of the non-zero spectrum in $P_{\ell G}^{r}$ with respect to that of $P_{G}^{r}$ for r≥0 suggests a link between both matrices to factorize and, consequently, between their respective embeddings. Therefore, uncovering this link allows us to understand (and then characterize) the structural differences between the respective latent spaces of nodes and edges (or even paths) for the same graph.

More precisely, the standard factorization approach for network embedding [36] states that the latent representations for nodes of G=(V,E) are obtained from the SVD of ${\hat{M}}_{G}=\log\left(\max\left(M_{G},1\right)\right)$, where $M_{G}$ is the Pointwise Mutual Information (PMI) matrix. More recently, Qiu et al. [15] showed that $M_{G}$ can be posed in the following terms:

(3) $$M_{G}=\frac{vol(G)}{b}S_{G},\;S_{G}=\left(\frac{1}{T}\sum_{r=1}^{T}P_{G}^{r}\right)D_{G}^{-1},\;$$

where $P_{G}=D_{G}^{-1}A_{G}$ is the transition matrix of *G*. This emphasizes the role of the random walks (RWs) in the resulting embedding. In particular, Qiu et al. show that the negative spectrum of $S_{G}$ is filtered out (zeroed) even for moderate values of the *window size T*. In practice, this leads to bound the singular values of $S_{G}$ by its eigenvalues (The formal proof is only correct when the *average matrix* $1/T(\sum_{r=1}^{T}P_{G}^{r})$ is symmetric.). Since the eigenvalues of $S_{G}$ decay exponentially, one should expect small optimal embedding dimensions for locally dense graphs, due to their small spectral gap [37,38]. In addition, the factorization of ${\hat{M}}_{G}$ provides a loss function (quality of the *K*-rank approximation) for quantifying the relative distortion of the current embedding with respect to that of an ideal one [39].

Herein, we extend the above analysis to link nodal embeddings with the embedding of higher-order entities (edges, paths, etc.). To that end, we compare the factorizaton of ${\hat{M}}_{G}$ with that of ${\hat{M}}_{\ell G}$ or ${\hat{M}}_{\ell^{p}G},p>1$. This is particularly interesting when considering directed networks (digraphs), where the following conditions apply: (a) We have a more flexible/general model; (b) There is solid mathematical machinery linking the ranks of *G*, ℓG, and $\ell^{p}G$ [28,31], as well as emphasizing the optimal diameter, degree preservation, and density of line graphs and their iterations [35,40].

**Figure 3:**
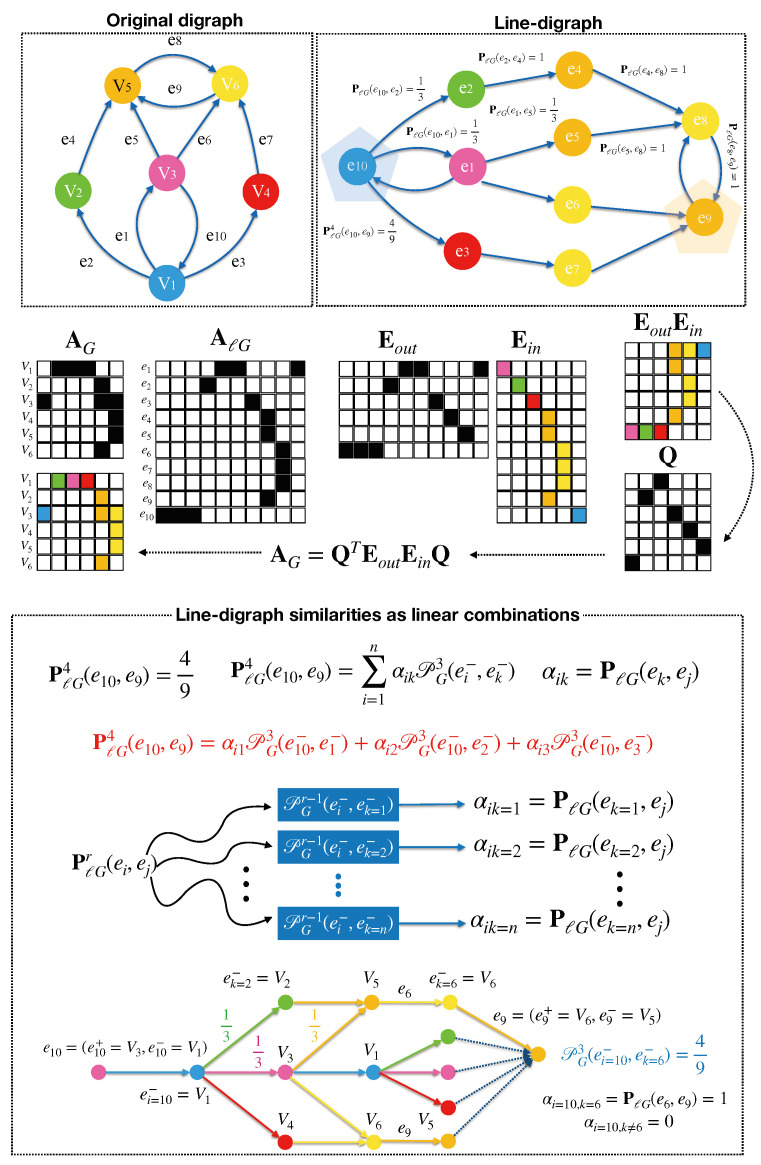
Explanation of the Linearity Theorem. (**Top**): Input digraph *G* and its line digraph ℓG. Colors index nodes as well as edges destinations. *Example*: compute $P_{\ell G}^{4}\left(e_{10},e_{9}\right)$, where $e_{10}$ is a hub and $e_{9}$ is an authority. (**Middle**): Adjacencies $A_{G}$ vs. $A_{\ell G}$. Clearly, $rank(A_{\ell G})=n$, with n=6 nodes in this case. $E_{out}$ retains the *n* different rows of $A_{\ell G}$. $E_{in}$ is the indicator matrix where $E_{in}\left(i,j\right)=1$ means that $e_{i}$ belongs to the class of equivalence j=1,…,n. Then, $E_{out}E_{in}$ is a permutation of $A_{G}$ (see colors to uncover the permutation matrix Q making $A_{G}=Q^{T}E_{out}E_{in}Q$). (**Bottom**): Explicit linear combinations. $P_{\ell G}^{r}$ as a function of $P_{G}^{r-1}$ (taking Q into account). $P_{\ell G}^{4}\left(e_{10},e_{9}\right)$ results from the aggregation of 3 paths in *G*, all of them starting from $e_{10}^{-}=V_{1}$ and ending at any node $e_{k}^{-}\in V$ in 3 steps. Two of the paths reach $V_{6}=e_{k=6}^{-}$ (and also $V_{5}=e_{k=9}^{-}$) and another one reachs $V_{5}=e_{k=9}^{-}$. Since only $e_{6}$ is linked with $e_{9}$, we only retain the scores of the paths reaching $V_{6}=e_{k=6}^{-}$, i.e., $P_{\ell G}^{4}\left(e_{10},e_{9}\right)=\frac{1}{3}+\frac{1}{9}=\frac{4}{9}$.

Here follows our main formal result in this paper. The intuition behind the following theorem is that random walks of length *r* in ℓG are linear combinations of n=|V| random walks in *G* of length r−1 for any length r>1 (see Figure 3).

**Theorem** **1** (Linearity)**.** *Embedding edges can be posed concerning the linear combination of node embeddings. For a strongly connected digraph G=(V,E), i.e., without sinks and sources, and with line digraph ℓG, we have the following relationship between edge similarity and node similarity:*

(4) $$P_{\ell G}^{r}\left(e_{i},e_{j}\right)=\sum_{k=1}^{n}\alpha_{ik}P_{G}^{r-1}\left(e_{i}^{-},e_{k}^{-}\right),\;\;\mathrm{with}\;\;\alpha_{ik}=P_{\ell G}\left(e_{k},e_{j}\right)\;,$$

*where $P_{\ell G}=D_{\ell G}^{-1}A_{\ell G}$ is the transition matrix of ℓG, $P_{G}$ is a permutation of $P_{G}=D_{G}^{-1}A_{G}$, the transition matrix of G and $e_{i}=\left(e_{i}^{+},e_{i}^{-}\right)$ denotes the directed edges (“out-of” and “into” components) of E (the nodes of ℓG).*

**Proof.**  If G=(V,E) is strongly connected, then we have that rank$(A_{\ell G})=n$, with n=|V| (see Lemma 4.2 in [28]). In addition, following the characterization theorem for the adjacency matrices of line graphs (Theorem 10 in [31]), we have that given two of the m=|E| rows of $A_{\ell G}$, they are either orthogonal or identical. As a result, there are only *n* different rows or *prototypes* in $A_{\ell G}$, say $e_{1},e_{2},\ldots ,e_{n}$, each one representing a *class of equivalence* (see Figure 3). Therefore, $A_{\ell G}$ can be factorized as follows: $A_{\ell G}=E_{in}E_{out}$, where $E_{in}$ is a m×n indicator matrix with $E_{in}\left(e_{i},e_{j}\right)=1$, if $e_{i}$ belongs to the class of $e_{j}$ and 0 otherwise; on the other hand, $E_{out}$ is the n×m matrix containing the different rows of $A_{\ell G}$ (characteristic patterns of linkage of the line graph).

Interestingly, the prototypes $e_{i},i=1,\ldots ,n$, are defined by the “into” node $e_{i}^{-}$ since edges ending in the same node $e_{i}^{-}$ in *G* exhibit the same pattern of linkage in ℓG. As a result, $A_{G}=Q^{T}E_{out}E_{in}Q$, where Q is a n×n permutation matrix where Q(i,j)=1 if $i=e_{j}^{-}$ and 0 otherwise, with i∈V. In addition, the transition matrices of both *G* and ℓG store the same probabilities in the non-zero positions and we have $P_{\ell G}=E_{in}E_{out}^{P}$ and $P_{G}=Q^{T}E_{out}^{P}E_{in}Q$, where $E_{out}^{P}\left(e_{i},e_{j}\right)=Pr\left[e_{i},e_{j}\right]$. Then, we have

(5) $$\begin{array}{ccc} P_{\ell G}^{r} & = & \underset{r\;\mathrm{terms}}{\underset{︸}{\left(E_{in}E_{out}^{P}\right)\left(E_{in}E_{out}^{P}\right)\ldots \left(E_{in}E_{out}^{P}\right)}}\\ & = & E_{in}\underset{r-1\;\mathrm{terms}}{\underset{︸}{\left(E_{out}^{P}E_{in}\right)\left(E_{out}^{P}E_{in}\right)\ldots \left(E_{out}^{P}E_{in}\right)}}E_{out}^{P}\\ & = & E_{in}\underset{r-1\;\mathrm{terms}}{\underset{︸}{\left(QP_{G}Q^{T}\right)\left(QP_{G}Q^{T}\right)\ldots \left(QP_{G}Q^{T}\right)}}E_{out}^{P}\\ & = & E_{in}P_{G}^{r-1}E_{out}^{P}\; \end{array}$$

where $P_{G}=QP_{G}Q^{T}$ is the permuted $P_{G}$. Then, we obtain $P_{\ell G}^{r}=E_{in}P_{G}^{r-1}E_{out}^{P}$, which is an m×m matrix. Since $E_{in}$ is a rank-*n* indicator matrix, the resulting $P_{\ell G}^{r}$ matrix has only *n* distinct rows, i.e., many edges have the same probabilistic pattern of linkage. This is why the n×m matrix $P_{G}^{r-1}E_{out}^{P}$ retains only the different rows of $P_{\ell G}^{r}$ and it is the matricial version of Equation (4). □

The proof of the linearity theorem uncovers the differences between the structure of $S_{G}$ (see Equation (3)) and that of $S_{\ell G}=\left(\frac{1}{T}\sum_{r=1}^{T}P_{\ell G}^{r}\right)D_{\ell G}^{-1}$. These differences lead to the rank preservation property of $S_{\ell G}$. This property is summarized by the following corollary:

**Corollary** **1** (Rank Preservation)**.** *The structure of $S_{\ell G}$ preserves better the rank than it does $S_{G}$ for values of T (window size) closer to the mixing time $t_{mix}$ of $P_{G}$.*

The proof is in Appendix A, where we refer the reader for a deeper understanding of the link between rank preservation and random walks.

### 2.5. Implications of Using Line Digraphs

In light of the formal analysis above, line digraphs and their embeddings have several pros and cons.

**Implications of Rank Preservation**. This is a key property for LDEs (Line Digraphs Embeddings) since the rank of the similarity matrix (and therefore that of the embedding) is closely related to the optimal dimension of the embedding. Following [41], a too-low dimensionality discards too much signal power, and this leads to a high bias (many vectors in the embedding collapse). On the other hand, a very large dimensionality leads to a high variance (the embedding becomes a noisy subspace). The optimal dimensionality is upper bound by the rank of the embedding. Thus, if we set a given dimensionality d≪n, LDEs (Line Digraph embeddings) hold a rank close to n−1, whereas DEs (Digraph embeddings) do not generally reach this rank. As a result, LDEs retain naturally the most informative part of the signal (which reduces the bias) with respect to DEs.

Finally, Theorem 1 and its corollary lead to the following definition of the component-wise similarity for the line digraph:

(6) $$M_{\ell G}\left(e_{i},e_{j}\right)=\frac{vol(\ell G)}{b\cdot d_{out}\left(e_{i}\right)}\left(\frac{1}{T}\sum_{r=1}^{T}\sum_{k=1}^{n}\alpha_{ik}P_{G}^{r-1}\left(e_{i}^{-},e_{k}^{-}\right)\right).$$

Therefore, factorizing ${\hat{M}}_{\ell G}$ allows us to classify edges in ℓG. Since herein the label of the edge is that of the destination node, we thus obtain a proxy to classify nodes in *G*. As a result, the differences in node-classification performance between ${\hat{M}}_{\ell G}$ and ${\hat{M}}_{G}$ are due to the following:

(a) The *flexibility of combining linearly many probabilities* (actually *n* terms) for defining a single probability of linking any pair of edges in the line digraph.
(b) The *better rank preservation* for the line digraph embedding with respect to its nodal counterpart. The rank of ${\hat{M}}_{\ell G}$ is O(n) even for large values of the window size *T*. Since edges with the same destination nodes are hashed to nearly the same embedding vector (see the proof of Theorem 1), this enforces the coherent classification of these destination nodes.

**Implications in Edge Mining**. As an additional result, the component-wise Katz similarity (used in HOPE [8]) with ℓG is $K_{\ell G}\left(e_{i},e_{j}\right)=\sum_{r=1}^{T}\beta^{r}\sum_{k=1}^{n}\alpha_{ik}A_{G}^{r-1}\left(e_{i}^{-},e_{k}^{-}\right)$, where $A_{G}$ is a permutation of $A_{G}$, and we down weight the number of paths between each pair of nodes in ℓG (edges in *G*). Such paths are linear combinations of the corresponding paths in *G*. This can be used to predict links in ℓG (second-order paths in *G*), to quantify the centrality of an edge (apply [42] to the line digraphs embeddings). We may also identify edge-hubs and authorities (applying HITS [43] to the line digraph, see Figure 3).

**The Need for Sparsification**. Computing the line digraph of a realistic (i.e., large) digraph is very challenging since we have $O(n^{2})$ nodes in the line digraph (the worst case arises with dense digraphs). To overcome this limitation, we must rely on *digraph sparsification* to *filter out non-critical edges* before embedding the line digraph. Although graph sparsification theory is mostly developed for non-directed graphs [44,45], there are recent approaches focused on asymmetric adjacency matrices, i.e., designed for digraphs. Herein, we follow Cohen et al.’s notion of matrix approximation: *two adjacency matrices $A_{\ell G}$ and ${\tilde{A}}_{\ell G}$ are similar if their symmetrizations hold good spectral properties. They are spectrally similar* [46]. With this notion to hand, we have that a good approximation (sparsification) ${\tilde{A}}_{\ell G}$ should preserve the in-degrees and out-degrees of $A_{\ell G}$ (Lemma 3.13 of [46]). As a result, the node e=(i,j) in ℓG (edges in G) will be sampled with probability $p_{e}$:

(7) $$p_{e}=\frac{1}{2n}\left[\frac{1}{d_{out}\left(i\right)}+\frac{1}{d_{in}\left(j\right)}\right]\;.$$

where $d_{out}\left(i\right)$ and $d_{in}\left(j\right)$ are, respectively, the out-degree of *i* and the in-degree of *j*. The probability of keeping the error of the approximation below ϵ with probability *p* is achieved with sampling independently $k\geq 128\cdot \left(2n/\epsilon^{2}\right)\log\left(2n/p\right)$ edges (see Theorem 3.9 in [46]).

In our experiments, we will analyze how competitive is the line digraph embedding in terms of classifying and clustering nodes with a decreasing number of sampled edges. Therefore, our approach is consistent with analyzing the robustness of the embedding to attacks (graph poisoning) [39]. Since edges are removed according to not linking good hubs with good authorities, both hubs and authorities are preserved as much as possible (hubs visit many authorities, and authorities are visited from many hubs). However, the singular gap (difference between the main singular value and the second best) may be reduced. Thus, we interpret the performance stability as evidence of the preservation of the singular gaps [47].

## 3. Experimental Results

### 3.1. Experimental Setup

In this paper, we analyze the following networks:

(a) Directed single-label citation networks: Cora [48]—Citation network containing 2708 scientific publications with 5278 links between them. Each publication is classified into one of 7 categories. CiteSeer for Document Classification [48]—Citation network containing 3312 scientific publications with 4676 links between them. Each publication is classified into one of 6 categories. Wiki—Contains a network of 2405 web pages with 17,981 links between them. Each page is classified into one of 19 categories. https://github.com/thunlp/MMDW/ (accessed on 10 March 2025). Citeseer and Cora have been retrieved from LINQS (https://linqs.soe.ucsc.edu/data, accessed on 10 March 2025). See Table 1 for details.

(b) Originally undirected multi-label networks: Protein-Protein Interactions (PPI)—Subgraph from PPI corresponding to Homo Sapiens. https://downloads.thebiogrid.org/BioGRID (accessed on 10 March 2025) [49]. Wikipedia Part-of-Speech (POS)—Co-occurrence of words appearing in the first million bytes of the Wikipedia dump. The categories correspond to the Part-of-Speech (POS) labels inferred by the Stanford POS-Tagger. Facebook social circles [50] that are treated as directed by adding bidirectional edges. These datasets have been retrieved from SNAP (https://snap.stanford.edu/node2vec/, accessed on 10 March 2025) [51]. See Table 2.

All the experiments were run on an Intel Xeon(R) W-2123 CPU @ 3.60 GHz ×8, equipped with a GeForce RTX 2080 Ti and 32 Gb RAM. Regarding the hyper-parameters of node2vec and NetMF, we have used window size T=10, rw-path length L=80, 10 walks per node, p=q=1 and dimension of the embedding vectors d=128. The down-weighting parameter for the HOPE algorithm is β=0.1. The obtained results are quite invariant to changes in these settings.

### 3.2. Classification Experiments

**Protocol**. The classification experiments are performed by training a logistic regression classifier with the embedding vectors corresponding to 50% of the nodes and tested with the remaining vectors, using the OpenNE framework (https://github.com/thunlp/OpenNE, accessed on 10 March 2025). Some cells in the results tables have been left blank due to computational limitations. Concerning the sparsification level, for the *inverse degree of sparsification* IDS z∈(0,1], we sample O(zm) edges with probability $p_{e}$ given in Equation (7). A descending IDS illustrates better than a pair ϵ,p the robustness of the embedding.

Then, we proceed to embed edges and use these embeddings for edge classification and/or clustering. Since edges inherit their labels from nodes, the latter process returns a proxy representation for node classification or clustering.

**Figure 4:**
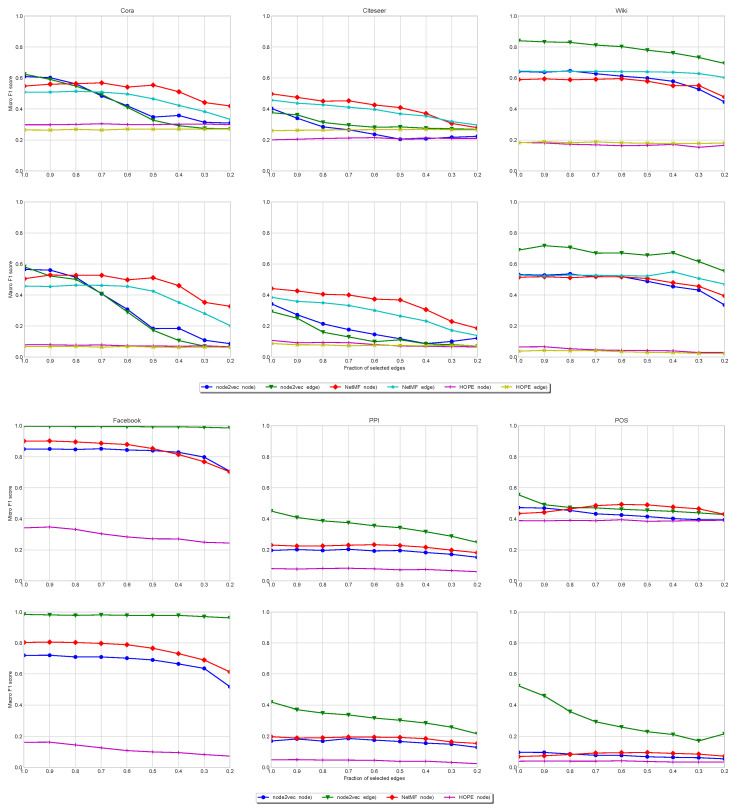
Classification stability. (**Top**): directed single-label citation networks. Legend: node2vec node refers to performance for the nodal embedding, node2vec edge refers to performance for edge embedding, and similarly for NetFM and HOPE. (**Bottom**): undirected multi-label networks. (**Top**): Micro-F1 score. (**Bottom**): Macro-F1 score.

**Citation Networks**. In Figure 4-Top, we show the stability of node classification for Cora, Citeseer, and Wiki. In all cases, the node and edge (line digraph) versions of NetMF are quite stable, even for small values of IDS (very sparse). However, the reachability patterns of Cora and Citeseer follow a power law with respect to the visiting of nodes in random walks (see Figure 1), which does not change when we apply the line graph (see their large numbers of sources and sinks in Table 2). Under these conditions, the random walks running on the line digraphs cannot reach many more different (contextual) nodes than when they run on the original graphs. This leads to very redundant embeddings. In addition, they have a low edge density. As a result, the LDE (node2vec(edge)) is not always the best alternative (especially in Citeseer).

However, for the Wiki network, the reachability pattern does not follow a power law (see Figure 1). In addition, the reachability pattern of the line digraph is even more entropic than that of the original graph. This is consistent with having much less sinks than Cora and Citeseer. It also has less sources than these networks but the higher density of the network allows the random walks exploring the line digraph to reach many different nodes. This leads to less redundant embeddings for the nodes of the line digraph. The node2vec(edges) algorithm works better because it reduces the redundancy with respect to the NetMF factorization.

In all cases, HOPE is stable but its performance is the lowest. It is only competitive with NetMF and node2vec in Cora and Citeseer because of their power laws. This is consistent with some findings showing that Katz similarity is competitive for networks with high-degree node coverage [52].

**Undirected Multi-label Networks**. Regarding PPI, Facebook, and POS, in Figure 4-Bottom, we show that in all cases the line digraph improves significantly the classification scores of the alternatives. It is worth mentioning that in all the cases in which the line digraph presents a significant improvement, it corresponds to datasets with a large edge/node ratio (i.e., cases in which the size of the line digraph presents a drastic growth with respect to the original graph). This is also the case of Wiki, in the citation networks.

**Stability Under Sparsification**. Concerning the citation networks, we do not observe significant differences between the magnitudes and the evolutions of their average singular gaps. However, Wiki has larger gaps than Cora and Citeseer for large values IDS (a small fraction of removed edges). Additionally, the most stable network (Facebook) has the largest average singular gap for the line digraph representation: 16.2 (line digraph) vs. 2.65 (original graph).

*Summarizing, the line digraph embedding improves the alternatives when it is possible to reduce the redundancy. This is explained by the rank property of the line digraph. In general, line digraphs are more robust than original graphs with respect to increasing levels of sparsification, especially when their average singular gap is large (Facebook).*

### 3.3. Clustering Experiments

**Protocol**. We evaluate the performance of the obtained embeddings for community detection problems, by applying agglomerative clustering on the embedding vectors. The total number of classes *C* is set to the number of labels. We analyze both the Adjusted Rand Index (ARI) and the modularity, which is defined for digraphs with adjacency matrix A, volume *W*, and proposed partition: P, as

(8) $$Q\left(A\right)=\frac{1}{W}\sum_{C\in P}\sum_{i,j\in C}\left[A_{ij}-\frac{d_{out}\left(i\right)d_{in}\left(j\right))}{W}\right]\;.$$

**Figure 5:**
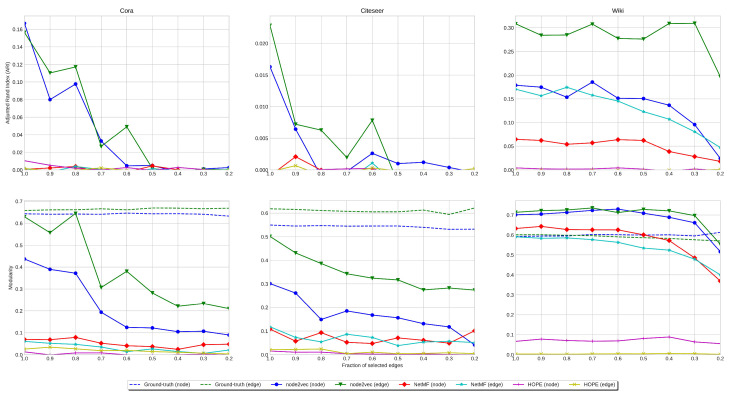
Clustering stability: Evolution of ARI (**top**) vs. evolution of modularity (**bottom**) for different sparsification levels (fraction of preserved edges). Legend: same as in classification results.

**ARI Analysis**. We run clustering experiments on all our single-label datasets: Cora, Citeseer, and Wiki. In Figure 5, we show the ARI values obtained by clustering the embedding of the original graph and the line digraph corresponding to the citation datasets. In the case of Cora, the results with the original graph, the line digraph, and the iterated line digraph are similar, but in the other cases, the line digraph outperforms the results obtained with the original graph.

**Modularity Analysis**. Regarding modularity, we have obtained both the modularity of the partition given by the original labeling of the graphs (ground truth), and the modularity of the partition provided by the clustering. Consistently with the ARI values, the modularity obtained with the line digraph outperforms the results of the original graph. In addition, we can observe that the modularity of the ground truth is almost the same in the original digraph and the line digraph. Interestingly, in Wiki we outperform the clustering obtained with the ground truth labeling.

**Stability under Sparsification**. The performance of clustering is less stable than that of classification for increasing levels of sparsification (decreasing IDS). This is due to the multi-modality of the labelled communities. *Summarizing, the performance of clustering is less stable than that of classification, but still the best results are obtained with the line digraph representation.*

### 3.4. GNN Experiments

To further validate our theoretical findings about line digraphs and their stability under sparsification, we extended our analysis to modern Graph Neural Networks (GNNs). While our previous experiments focused on node2vec-like embeddings, here we evaluate whether the maximum entropy properties of line digraphs translate to improved performance with GNN architectures as well.

GNNs have emerged as powerful tools for learning graph-structured data by iteratively updating node representations through neighborhood aggregation schemes [1]. In their simplest form, vanilla GNNs follow a message-passing framework where each node aggregates feature vectors from its neighbors and then applies a learnable transformation to update its own representation [53,54]. This process can be expressed as follows:

$$h_{v}^{\left(l+1\right)}=\sigma \left(W^{\left(l\right)}\cdot \mathrm{AGGREGATE}\left(\{h_{u}^{\left(l\right)}:u\in N\left(v\right)\}\right)\right)$$

where $h_{v}^{\left(l\right)}$ is the feature vector of node *v* at layer *l*, N(v) represents the neighbors of *v*, $W^{\left(l\right)}$ is a learnable weight matrix, and σ is a non-linear activation function.

We evaluated four state-of-the-art GNN architectures [55] that extend this basic framework in different ways:

1. **Graph Convolutional Networks (GCN)** [1]: Uses a simple weighted average of neighbor features with spectral motivation.
2. **Graph Attention Networks (GAT)** [2]: Employs attention mechanisms to weight neighbor contributions differently.
3. **GraphSAGE (SAGE)** [4]: Samples a fixed number of neighbors and learns how to aggregate their features.
4. **Approximate Personalized Propagation of Neural Predictions (APPNP)** [3]: Separates feature transformation from propagation using personalized PageRank.

Following standard practice in the literature, we used the established split of 60% training, 20% validation, and 20% testing sets for all datasets. For each architecture and dataset, we implemented two different sparsification strategies:

- **Edge sparsification**: Randomly sampling edges while maintaining graph connectivity.
- **Node sparsification**: Selecting a subset of nodes and preserving their associated edges.

The fraction of selected edges varied from 0.0 to 1.0 in steps of 0.1, allowing us to assess model robustness across different sparsification levels.

**Figure 6:**
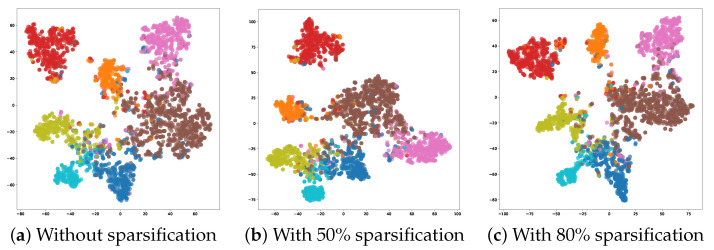
Visualization of learned edge embeddings using t-SNE dimensionality reduction of the Cora dataset. Colors represent different ground truth classes. Note that even with significant sparsification (80% of edges removed), the embedding space maintains clear community structures and distinct class boundaries, demonstrating the entropy-preserving properties of line digraphs even under aggressive edge reduction.

Figure 6 visualizes the learned edge embeddings from the line digraph of Cora using t-SNE dimensionality reduction at different sparsification levels. Remarkably, even with significant sparsification (80% of edges removed), the embedding space preserves clear community structures with well-defined boundaries between different classes. This visual evidence supports our theoretical claim that line digraphs maintain high entropy between communities even under aggressive sparsification, preserving the essential topological structure needed for node classification.

**Figure 7:**
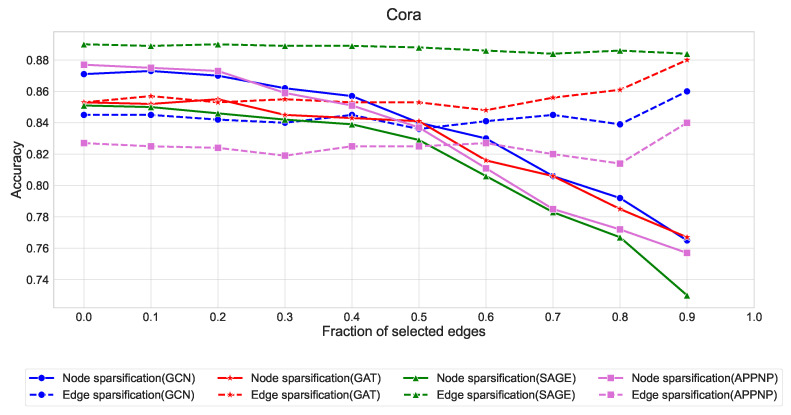
Comparison of node and edge sparsification strategies across GNN architectures on the Cora dataset. Solid lines represent node sparsification, dashed lines represent edge sparsification. The x-axis indicates the fraction of edges preserved (from 0.0 to 1.0), while the y-axis shows classification accuracy. Edge sparsification consistently outperforms node sparsification, with GraphSAGE (dashed green) showing remarkable stability even with minimal edge retention.

**Figure 8:**
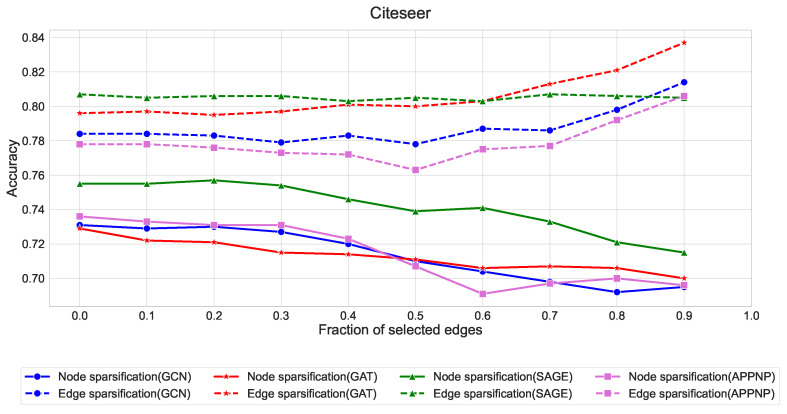
Comparison of node and edge sparsification strategies across GNN architectures on the Citeseer dataset. Solid lines represent node sparsification, dashed lines represent edge sparsification. The performance gap between edge and node sparsification is more pronounced in Citeseer than in Cora, with edge-based methods (dashed lines) maintaining consistently higher accuracy across all sparsification levels. This suggests that preserving edges is particularly important for maintaining the structural information in citation networks with sparse connectivity patterns.

**Figure 9:**
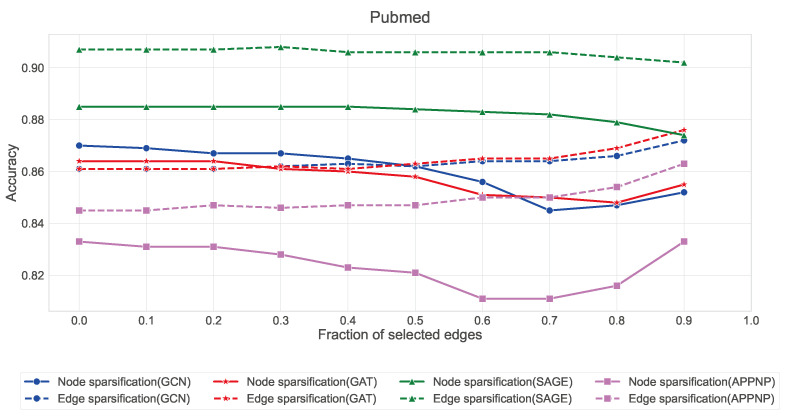
Comparison of node and edge sparsification strategies across GNN architectures on the Pubmed dataset. Solid lines represent node sparsification, dashed lines represent edge sparsification. All GNN architectures show remarkable stability under edge sparsification (dashed lines), with GraphSAGE maintaining above 90% accuracy even with only 10% of edges preserved. This demonstrates that strategic edge preservation captures the essential topology of the graph, aligning with our theoretical findings about the maximal entropy properties of line digraphs.

The quantitative results in Figure 7, Figure 8 and Figure 9 reveal several key findings:

1. Edge sparsification consistently outperforms node sparsification across all three datasets, with the effect being most pronounced in Citeseer and Pubmed. This aligns with our theory that strategic edge sampling preserves more of the graph’s spectral properties than node-based approaches.
2. GraphSAGE exhibits remarkable stability under edge sparsification, maintaining accuracy above 88% in Cora, 80% in Citeseer, and 90% in Pubmed even with only 10% of edges. This robustness can be attributed to its neighborhood sampling strategy, which naturally accommodates sparser structures.
3. GCN and GAT show similar patterns, with edge sparsification providing better performance than node sparsification when the fraction of selected edges is below 0.5.
4. APPNP demonstrates higher sensitivity to sparsification compared to other architectures, suggesting its personalized PageRank-based propagation mechanism benefits from denser graph structures.

These findings complement our earlier analysis of node2vec-like embeddings, providing additional evidence that the line digraph representation can maintain high classification performance even under significant edge reduction. The consistency of these results across both traditional embedding approaches and modern GNN architectures validates our theoretical framework regarding the entropy-maximizing properties of line digraphs and their stability under sparsification.

## 4. Conclusions

In this paper, we have proposed an edge-centric embedding: embedding the line digraph instead of the original digraph. Our motivation is that node-centric approaches do not model edge directionality. We have uncovered a formal link between the embedding of edges and that of nodes. This link leads to the ranking property, which allows us to (a) better classify and cluster nodes of the original graph, when we use their respective directed-edges embeddings as proxies, and (b) better understand the latent spaces of both nodes and edges, which may explain the success of their co-embedding. In addition, we filter out in advance the non-critical edges of the original graph to make the line digraph computation scalable. Our experiments show that line digraphs are stable under increasing levels of sparsification. These experiments are performed from two different angles: for node2vec-like latent spaces, where our nodes do not have attributes (only topology), and GNNs, where each node is attributed and the combination between topology and aggregation determines the latent spaces. In both cases, the entropy-maximization margin of line graphs is key.

Future work is motivated by the scalability problems arising in dense graphs, but this is inherent to investigating the role of an edge in a graph. This requires the development of more powerful and even *learnable* sparsifiers. In addition, the rank-preservation property (which is congruent with determinantal point processes) suggests that edge-embeddings could be more powerful than node-based ones for classifying graphs themselves through deep sets [56]. Finally, we will test to what extent LDEs contribute to structural transfer learning [26].

## Figures and Tables

**Table 1:** Properties of Cora, Citeseer, and Wiki datasets.

Dataset	Nodes	Edges	Classes	Sinks	Sources	Ratio	Density
Cora	2708	5429	7	486	1143	2.004801	0.0741%
Cora line graph	5429	10,147	7	716	1926	1.869037	0.0344%
Cora iterated	10,147	18,678	7	1213	3358	1.840741	0.0181%
Citeseer	3327	4732	6	1006	1365	1.422302	0.0428%
Citeseer line graph	4732	6413	6	864	1332	1.355241	0.0286%
Citeseer iterated	6413	8254	6	894	1537	1.287073	0.0201%
Wiki	2405	16,523	17	19	360	6.870270	0.2858%
Wiki line graph	16,523	227,131	17	86	1242	13.746354	0.0832%
Wiki iterated	227,131	3,786,444	17	993	16,021	16.670749	0.0073%

**Table 2:** Properties of PPI, POS, and Facebook datasets.

Dataset	Nodes	Edges	Classes	Diameter	Ratio	Density
PPI	3890	76,584	50	8	19.687404	0.5062%
PPI line graph	76,584	6,113,024	50	9	79.821164	0.1042%
Facebook	4039	176,468	10	8	21.845506	0.5410%
Facebook line graph	176,468	18,806,166	10	9	106.569837	0.0604%
POS	4777	184,812	40	3	38.687879	0.8100%
POS line graph	184,812	99,322,576	40	4	537.424929	0.2908%

## Data Availability

The raw data supporting the conclusions of this article will be made available by the authors on request.

## Abbreviations

The following abbreviations are used in this manuscript:

LDE	Line Digraphs Embeddings
PMI	Pointwise Mutual Information
PPI	Protein-Protein Interactions
POS	Part-of-Speech
RW	Random Walk
SBM	Stochastic Block Model
HITS	Hyperlink-Induced Topic Search
IDS	Inverse Degree of Sparsification
ARI	Adjusted Rand Index

## Appendix A. Proof of the Rank-Preservation Corollary

**Corollary** **A1** (Rank Preservation)**.** *The structure of $S_{\ell G}$ preserves better the rank than it does $S_{G}$ for values of T (window size) closer to the mixing time $t_{mix}$ of $P_{G}$.*

**Proof.** From the definition of $S_{G}$ we have the following:

(A1) $$\lim_{T\to \infty }S_{G}=\lim_{T\to \infty }\left(\frac{1}{T}\sum_{r=1}^{T}P_{G}^{r}\right)D_{G}^{-1}=\Pi_{G}D_{G}^{-1}\;,$$

where $\Pi_{G}$ is an n×n matrix whose rows repeat *n* times the steady state $\pi_{G}=\left[{\pi_{G}}_{1},{\pi_{G}}_{2},\ldots ,{\pi_{G}}_{n}\right]$ of the Markov chain defined by $P_{G}$. Therefore, the asymptotic rank of $S_{G}$ is one. Similarly, for $S_{\ell G}$ since we have the following:

(A2) $$\lim_{T\to \infty }S_{\ell G}=\lim_{T\to \infty }\left(\frac{1}{T}\sum_{r=1}^{T}P_{\ell G}^{r}\right)D_{\ell G}^{-1}=\Pi_{\ell G}D_{\ell G}^{-1}\;,$$

where the m×m matrix $\Pi_{\ell G}$ has *m* identical rows with the stationary state vector $\pi_{\ell G}=\left[{\pi_{\ell G}}_{1},{\pi_{\ell G}}_{2},\ldots ,{\pi_{\ell G}}_{m}\right]$. However, a closer look to the above limit uncovers an interesting property for the ℓG similarity:

(A3) $$\begin{array}{ccc} \lim_{T\to \infty }S_{\ell G} & = & \lim_{T\to \infty }\left(\frac{1}{T}\sum_{r=1}^{T}P_{\ell G}^{r}\right)D_{\ell G}^{-1}\\ & = & \lim_{T\to \infty }\left(\frac{1}{T}\sum_{r=1}^{T}E_{in}P_{G}^{r-1}E_{out}^{P}\right)D_{\ell G}^{-1}\\ & = & E_{in}\left[\lim_{T\to \infty }\left(\frac{1}{T}\sum_{r=1}^{T}P_{G}^{r-1}\right)\right]E_{out}^{P}D_{\ell G}^{-1}\\ & = & E_{in}\left[\lim_{T\to \infty }\frac{1}{T}\left(I+P_{G}+P_{G}^{2}+\ldots \right)\right]E_{out}^{P}D_{\ell G}^{-1}\;. \end{array}$$

Equation (A3) leads to Equation (A2), thus uncovering $\Pi_{\ell G}\approx E_{in}{\tilde{\Pi }}_{G}E_{out}^{P}$, where ${\tilde{\Pi }}_{G}=Q^{T}\Pi_{G}Q$, i.e., it is a permutation of $\Pi_{G}$ (see the proof of Theorem 1). However, for large but finite values of $T=T_{l}$, we have that

(A4) $$\begin{array}{ccc} P_{G}+P_{G}^{2}+\ldots +P_{G}^{T_{l}} & = & \left[I+P_{G}+P_{G}^{2}+\ldots +P_{G}^{T_{l}-1}\right]P_{G}\\ & = & \left[\left(P_{G}^{T_{l}}-I\right){\left(I-P_{G}\right)}^{-1}\right]P_{G}\;, \end{array}$$

For $T=T_{l}$, $S_{\ell G}$ is defined as

(A5) $$\begin{array}{ccc} S_{\ell G}^{large} & = & E_{in}\left[\frac{1}{T_{l}}\left(I+P_{G}+P_{G}^{2}+\ldots +P_{G}^{T_{l}-1}\right)\right]E_{out}^{P}D_{\ell G}^{-1}=E_{in}\left[\frac{1}{T_{l}}\left(I+\left(P_{G}^{T_{l}-1}-I\right){\left(I-P_{G}\right)}^{-1}P_{G}\right)\right]E_{out}^{P}\\ & = & \frac{1}{T_{l}}\left(E_{in}E_{out}^{P}+E_{in}\left[\left(P_{G}^{T_{l}-1}-I\right){\left(I-P_{G}\right)}^{-1}P_{G}\right]E_{out}^{P}\right)D_{\ell G}^{-1} \end{array}$$

(A6) $$\begin{array}{ccc} \; & = & \frac{1}{T_{l}}\left(E_{in}E_{out}^{P}+E_{in}\left[{\left(I-P_{G}\right)}^{-1}\left(P_{G}^{T_{l}-1}-I\right)P_{G}\right]E_{out}^{P}\right)D_{\ell G}^{-1}\;. \end{array}$$

However, the shape of $S_{G}^{large}$ is quite different:

(A7) $$S_{G}^{large}=\frac{1}{T_{l}}\left[{\left(P_{G}-I\right)}^{-1}\left(P_{G}^{T_{l}}-I\right)P_{G}\right]D_{G}^{-1}\;.$$

In terms of rank, for $T_{l}$ large enough (e.g., close to the mixing time $t_{mix}$), the ergodicity theorem leads to $P_{G}^{T_{l}}=\Pi_{G}$. In this case, $rank(S_{G}^{large})<n$, despite ${\left(P_{G}-I\right)}^{-1}$ exists (full rank), since we have $det\left(\left(P_{G}^{T_{l}}-I\right)P_{G}\right)=det\left(P_{G}^{T_{l}}-I\right)=0$. However, this is not (necessarily) the case of $S_{\ell G}^{large}$, despite ${\left(I-P_{G}\right)}^{-1}\left(P_{G}^{T_{l}-1}-I\right)P_{G}$ is not full rank. Since, $E_{in}E_{out}^{P}=P_{\ell G}$ (by definition) we have that $rank(E_{in}E_{out}^{P})=n$. Considering the worst case, where the rank of ${\left(I-P_{G}\right)}^{-1}\left(P_{G}^{T_{l}-1}-I\right)P_{G}$ is one, we have that post-multiplying this matrix by $E_{out}^{P}$ cannot increase this rank. In addition, the role of the pre-multiplying (indicator) matrix $E_{in}$ is just to finally produce a m×m matrix. Thus, in the worst case, $S_{\ell G}^{large}$ becomes the sum of a full-rank matrix and a rank-one matrix. Therefore, $rank(S_{\ell G}^{large})\geq n-1$. In this case, $S_{\ell G}^{large}$ is only full rank when the Sherman–Morrison conditions are fulfilled. In practice, however, $T_{l}\ll t_{mix}$, and one can (a priori) expect a better rank preservation behavior of $S_{G}^{large}$ with respect to $S_{\ell G}^{large}$. However, this is not always necessarily true. A close look to recent bounds of $t_{mix}$ in (Eulerian) digraphs [57] shows that if we define

(A8) $$t_{mix}\left(\epsilon \right)=\min\left\{t\geq 0:\;max_{x,y}\left|\frac{P_{G}^{t}\left(x,y\right)}{\pi_{G}\left(y\right)}-1\right|\leq \epsilon \right\}\;,$$

then the upper bound of $t_{mix}$ is O(mn). As a result, it is expected that $S_{\ell G}^{large}$ preserves its rank better than $S_{G}^{large}$ when *G* is dense. □
